# A small pilot trial of virtual group cognitive behavioral therapy for insomnia in gynecologic oncology patients

**DOI:** 10.1016/j.gore.2026.102152

**Published:** 2026-06-20

**Authors:** Abigail Low, Jena Cooreman, Michelle L. Dossett, Katherine Van Deventer, Matthew Ponzini, Machelle Wilson, Codi Cole, Rachel Ruskin, Gary Leiserowitz, Rebecca Brooks, Nancy Nguyen, Melissa Pham, Hui Chen

**Affiliations:** a University of California, Davis, School of Medicine, Sacramento, CA, United States of America; b University of California, Davis Comprehensive Cancer Center, Supportive Oncology and Survivorship Services, Sacramento, CA, United States of America; c University of California, Davis, Department of Internal Medicine, Sacramento, CA, United States of America; d Valley View Hospital, Department of Obstetrics and Gynecology, Glenwood Springs, CO, United States of America; e University of California, Davis, Division of Biostatistics, Department of Public Health Sciences, Sacramento, CA, United States of America; f University of California, Davis, Department of Obstetrics and Gynecology, Gynecologic Oncology, Sacramento, CA, United States of America

**Keywords:** Insomnia, Sleep wake disorders, Female genital neoplasms, CBT-I, Feasibility studies

## Abstract

**Objective:**

To evaluate the feasibility and acceptability of virtual, small-group cognitive behavioral therapy for insomnia (CBT—I) in patients with gynecologic cancers.

**Methods:**

Patients with gynecologic cancers were recruited to participate in a weekly, online, small-group CBT-I program. Eligible participants were ≥ 18 years old, English-speaking, had access to the internet, had moderate-severe insomnia (Insomnia Severity Index (ISI) score of ≥15), and were not taking prescription sleep medications. Feasibility was assessed by attendance, internet connectivity, and participation. An acceptability survey was administered at the end of each session. Exploratory endpoints included quality of life and insomnia symptoms. Participants tracked sleep outcomes using the Insomnia Coach Explorer. Follow-up assessments were completed 4, 12, and 24 weeks following program completion.

**Results:**

Five participants were recruited and four completed the intervention. 60% of participants attended four or more sessions. Internet connectivity, participation, and acceptability met the predefined benchmarks for feasibility. Compared to a baseline score of 18, mean ISI scores decreased by more than 6 points by week 6 (10.81, 95% CI: 7.12–14.5, *p* = 0.0002). The improvement in ISI scores was sustained throughout the follow-up period of 24 weeks. There was a decrease in the number of times awoken from the baseline 2.10 (−0.21/week, *p* = 0.021). No significant changes were observed in PSQI or FACT-G7 scores or other sleep outcomes from the sleep diary.

**Conclusions:**

Virtual, small-group CBT-I is a feasible and acceptable strategy for patients with gynecologic cancers to manage insomnia. Implementation of this intervention may also help expand access to evidence-based treatment for insomnia.

## Highlights

• Insomnia is highly prevalent and understudied in gynecologic oncology patients.
• Virtual, group-based CBT-I is a feasible and acceptable approach to insomnia.
• Insomnia severity and number of times woken improved with the intervention.
• This model may improve access to insomnia therapy for gynecologic cancer patients.

## 1 Introduction

Insomnia, characterized by difficulty falling or staying asleep and poor sleep quality, is a common and life-altering symptom in patients with cancer (Buysse, 2013). Up to 88% of patients with cancer experience insomnia at the time of their diagnosis, which is significantly higher than the 10–20% prevalence in the general population (Savard and Morin, 2001; Woodward, 2011; Buysse, 2013). Insomnia often persists even after completion of cancer treatment, with 23–44% of cancer survivors continuing to experience insomnia years after remission (Savard and Morin, 2001). Compared to other cancer populations, patients with gynecologic cancers have one of the highest prevalences of sleep disturbance, with nearly one-third reporting insomnia (Davidson et al., 2002; Fiorentino et al., 2010; Savard et al., 2014; Cai et al., 2025). Contributing risk factors include older age, female sex, poor physical health, and cancer-related pain (Taylor et al., 2005; Morin et al., 2006; LeBlanc et al., 2007). Beyond its high prevalence, insomnia can lead to substantial morbidity. Among patients with gynecologic cancers, insomnia has been associated with increased fatigue, depression, anxiety, functional impairment, and poor quality of life (Clevenger et al., 2013; Xie et al., 2025). As such, effective management of insomnia may represent an important opportunity to reduce symptom burden and improve well-being among patients with gynecologic cancers. Cognitive behavioral therapy for insomnia (CBT—I) is the gold-standard, non-pharmacologic treatment for insomnia (Woodward, 2011; NCCN Survivorship Guidelines [Internet], 2026). CBT-I approaches insomnia in a multimodal fashion, incorporating behavioral interventions such as stimulus control therapy and relaxation training, in addition to cognitive restructuring (Morin et al., 1993). It also uses educational strategies to help patients improve their sleep hygiene and sleep environment (Aricò et al., 2016). Multiple studies have consistently shown the efficacy of CBT-I in managing insomnia in patients with cancer without the risk of adverse events associated with pharmacologic therapies, such as benzodiazepines, sedatives, and tricyclic antidepressants (Fiorentino et al., 2010; Hofmann et al. 2012; Garland et al., 2014; Johnson et al., 2016). Many professional societies recommend CBT-I for initial insomnia management (Schutte-Rodin et al., 2008; Brasure et al., 2016; Qaseem et al., 2016; Riemann et al., 2017). Specifically for patients with cancer, the National Comprehensive Cancer Network (NCCN) deems CBT-I as the preferred treatment for cancer-related insomnia (NCCN Survivorship Guidelines [Internet], 2026).

CBT-I is usually conducted as 50–90-min sessions over the course of 5–8 weeks (Bastien et al., 2004; Savard et al., 2005, Savard et al., 2014; Matthews et al., 2014; Garland et al., 2014). Since CBT-I is traditionally performed by trained professionals in an in-person, one-on-one setting, access to care is limited by the number of mental health professionals (Savard et al., 2014). Since the COVID-19 pandemic, internet-based methods have helped increase access for individuals in resource-limited areas. In patients with breast cancer, live internet-based CBT-I has improved insomnia symptoms compared to waitlist controls (Garland et al., 2024). Although patients with breast and gynecologic cancers share some overlap, there are enough distinct differences in demographics, treatment regimens and disease course that warrant dedicated investigation in a gynecologic cancer population. For gynecologic malignancies, only an in-person, individualized format for CBT-I has shown to improve sleep efficiency, total wake time, and sleep latency compared to the psychoeducation alone (Padron et al., 2022). To date, no study has evaluated live, small-group, virtual CBT-I in patients with gynecologic cancers.

The objectives of this study were to assess the feasibility and acceptability of delivering CBT-I in a small-group, live, virtual format to patients with gynecologic cancers, and to evaluate patient-reported outcomes (PROs) for insomnia, sleep quality, sleep metrics, and quality of life. Demonstrating feasibility of this approach is a first step to improving access to this guideline-recommended insomnia treatment.

## 2 Methods

This was a pilot study aimed at evaluating the feasibility and acceptability of delivering CBT-I in a virtual, small group format to patients undergoing treatment or surveillance for gynecologic cancer at the UC Davis Health (UCDH) Comprehensive Cancer Center. This study was approved by the UCD Institutional Review Board. All participants provided written informed consent prior to participation.

### 2.1 Participants

Patients with a history of any gynecologic cancer and who were English-speaking were screened for eligibility during the enrollment period. Other inclusion criteria were age greater than 18 years old, access to the internet, the ability to complete questionnaires online through the UC Davis Research Electronic Data Capture (REDCap) secure server, the ability to download the Insomnia Coach Explorer App on their personal device, and had moderate to severe insomnia as defined by an Insomnia Severity Index (ISI) score of ≥15. Participants could be currently in surveillance or undergoing active cancer treatment. Patients with a remote history of insomnia but without active symptoms, who were taking prescription sleep medication or had other reasons for poor sleep (e.g. obstructive sleep apnea, chronic obstructive pulmonary disease, mood disorders), and without the ability to connect with both audio and video onto the secure Cisco WebEx platform were excluded.

### 2.2 Recruitment

Participants were recruited over a two-month period through a two-step process. During the first step, potentially eligible patients were identified using the electronic medical record (EMR), direct provider referrals from the UC Davis Health Comprehensive Cancer Center gynecologic oncology clinic, and the UC Davis StudyPages website. Any potentially eligible patients identified using EMR were sent an invitation to participate. Interested patients and those referred by their provider were called and screened for eligibility. Preliminarily eligible participants signed written informed consent and underwent a second screening step to determine eligibility for group therapy. They were contacted by a CBT-I professional to discuss the program, goals of therapy, and guidelines for group participation over the phone. Participants then verbally consented to participate in group therapy.

### 2.3 Assessments

Prior to the intervention, participants completed the following PRO measures: ISI, Pittsburgh Sleep Quality Index (PSQI) and the Functional Assessment of Cancer Therapy General 7-Item Version (FACT-G7) to characterize baseline sleep characteristics and general quality of life. Additionally, participants were asked to download the Insomnia Coach Explorer App on their mobile device or tablet. Insomnia Coach Explorer is a free application created and supported by the 10.13039/100000738Department of Veterans Affairs (VA), which can be used as a sleep diary.

During the intervention and follow-up period, participants completed the ISI, FACT-G7, and PSQI assessments. At the end of each weekly session, participants also completed a 3-question survey regarding the acceptability of the intervention's virtual, small-group format. The second and third questions were open-ended, inquiring about what participants liked about the session and what could have been improved.

### 2.4 Intervention

Participants completed the 6-session, weekly, online small-group CBT-I program, which taught and provided opportunities to practice key sleep improvement strategies such as sleep logging, sleep restriction, stimulus control, sleep hygiene, sleep scheduling, cognitive restructuring, anxiety management, and relaxation techniques (Morin et al., 1993). Weekly sessions also included time to problem-solve any challenges each participant had with implementing these strategies. Each session was 90 min in duration, administered via Cisco WebEx, and attended by the CBT-I professional who facilitated the session, the research team, and the participants. The CBT-I professional is a Licensed Clinical Social Worker and Certified Oncology Social Worker with seven years of experience practicing CBT-I with adult cancer patients.

Under the guidance of the CBT-I professional, participants reviewed their sleep diaries, calculated their sleep efficiencies, and modified their sleep schedules based on their progress each week. During each session, two members of the research team observed the session and collected data on participant attendance, engagement, and issues with internet connectivity. Following each session, participants completed the ISI and FACT-G7 assessments, as well as the acceptability survey on REDCap. During the follow-up phase, participants were asked to complete the ISI, PSQI, and FACT-G7 assessments at 4 weeks, 12 weeks, and 24 weeks after program completion.

## 3 Statistical Considerations.

### 3.1 Sample Size

Since this was a pilot feasibility study, sample size calculations based on power were not performed. We aimed to recruit a maximum of 12 subjects to allow for attrition. In previously described studies, small group CBT-I has been performed in groups of 2–10 individuals (Morin et al., 1993; Espie et al., 2007; Epstein and Dirksen, 2007; Blom et al., 2015). However, most commonly, group sizes included 6 participants (Morin et al., 1993; Espie et al., 2007; Epstein and Dirksen, 2007; Blom et al., 2015).

### 3.2 Feasibility

The main prespecified feasibility metric was attendance. More than half of the participants needed to attend at least 4 out of the 6 sessions to gain benefit from the program. Therefore, the rate of attendance among all possible sessions needed to be 67% or greater to be considered feasible.

Other feasibility metrics included assessing issues related to internet connectivity and other technical issues that arose due to the remote video nature of the sessions. Missed sessions due to internet connectivity and other technical problems were recorded. If participants missed >50% of any given session, that session was considered a missed attendance.

Finally, all subjects were required to participate at least once during each session. Participation included verbal and written questions or comments to the group. Comments or questions directed privately at the research staff were not counted as group participation. Attendance was reported on an individual participant level as a percent of sessions attended over total sessions using mean and range. Attendance was reported on a group level, percent of individuals who attended four or more sessions using mean and range.

### 3.3 Acceptability

Participants were surveyed at the end of each session to assess the acceptability of this virtual small group session format. A session was deemed acceptable if 50% or more of the participants who attended rated it as acceptable. The proportion of participants who deemed each session acceptable was estimated using mean and range.

### 3.4 Secondary endpoints

Secondary endpoints included insomnia symptoms based on ISI and PSQI responses, quality of life based on FACT-G7 responses, and different characteristics of sleep quality, which were collected from the sleep diary using the Insomnia Coach Explorer App data. The aspects of sleep that were evaluated include total sleep time (TST), wake after sleep onset (WASO), number of times awoken, sleep latency, and sleep efficiency ([total sleep time/time in bed] x 100). These sleep and quality of life secondary endpoints were displayed graphically as trends over time. Using a linear mixed effects model, insomnia, quality of life, and sleep aspects at baseline, at the completion of the intervention, and after the follow-up period were compared. This study was not designed to evaluate treatment effect, but rather to gather information about the variance in the data and feasibility of these study procedures. While insomnia may improve (or worsen) with cessation of cancer treatment, controlling for possible confounding factors over the course of recovery would require a control group and is beyond the scope of this feasibility study. However, this study can help guide the design of future randomized clinical trials.

## 4 Results

### 4.1 Recruitment and participants

**Fig. 1:**
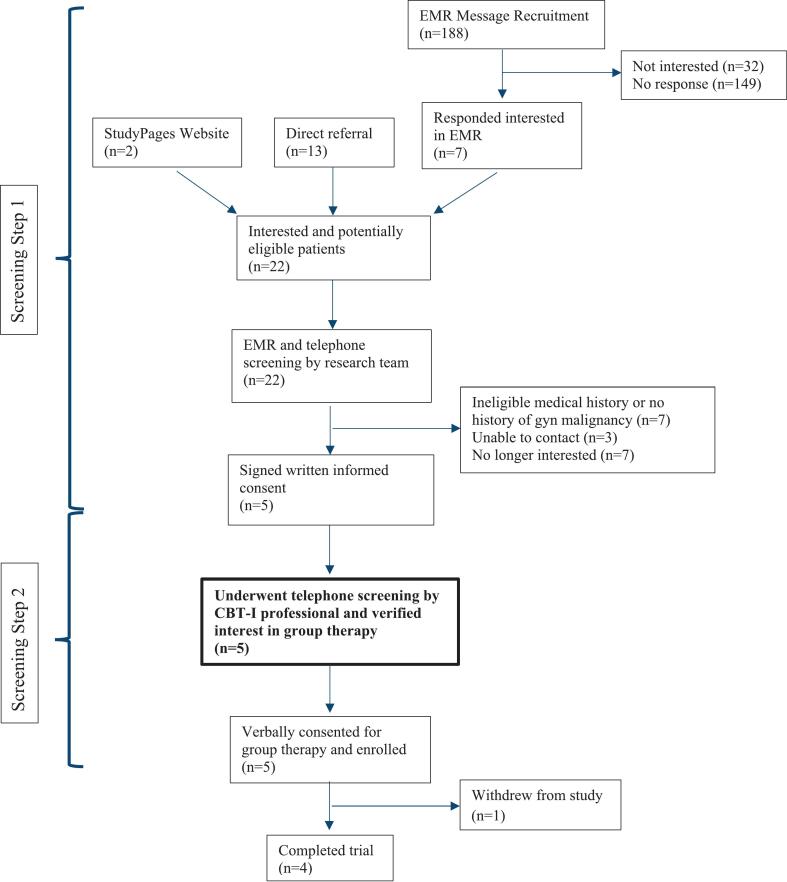
Trial Enrollment Caption: A two-step screening process was used for enrollment. In the first step, participants were recruited and underwent usual screening by the research team. Participants then had a second telephone-based screening by the CBT-I professional to confirm interest in group therapy.

Between January 2024 and March 2024, 209 patients who fit the eligibility criteria of having a history of gynecologic malignancy based on ICD-10 codes, being English-speaking, being 18 years old or older, and having an appointment in the gynecologic oncology clinic were identified using an electronic medical record (EMR) search. Of these, 188 patients had active MyChart access and could be sent an electronic invitation; the remaining 21 lacked an active patient-portal account and could not be contacted by this method. These 188 patients were sent an initial EMR invitation to participate: 149 did not respond, 32 responded but declined to participate, and 7 responded and expressed interest. Additional recruitment methods included direct referrals from clinic providers (*n* = 13) and the study website (*n* = 2), yielding 22 interested and potentially eligible individuals. These 22 individuals entered a two-step screening process. Ultimately, five eligible participants were consented and enrolled into the trial. Four completed the trial and one withdrew from the study after session 2 (Fig. 1).

Patient demographics are displayed in Table 1. The mean age was 57.6 years old. Most participants were white (80%). The types of cancer represented in this study included ovarian/fallopian tube/primary peritoneal (60%), endometrial (20%), and carcinoma of Mullerian origin (20%). All patients were diagnosed with advanced disease and had undergone systemic therapy. 60% had a history of recurrent disease, of those 66.7% were in remission and 33.3% were on active treatment. Overall, 60% were on active treatment at the time of intervention, with the remainder receiving primary systemic therapy. 80% had undergone prior surgery and 40% had received radiation.

**Table 1:** Characteristics of Participants (*N* = 5).

Age (years)^1^	57.6 (43–70)
Race	White/Caucasian – 80%
	Other – 20%
Ethnicity	Not Hispanic/Latino – 100%
Cancer Type	Ovarian, Fallopian Tube, Peritoneal – 60%
	Endometrial – 20%
	Mullerian Origin – 20%^2^
Stage at Diagnosis	III – 80%
	IV – 20%
History of Recurrent Disease	Yes – 60%
	No – 40%
Currently on Treatment	Yes – 60%
	No – 40%
Prior Surgery	Yes – 80%
	No – 20%
Prior Systemic Therapy	Yes – 100%
Prior Radiation	Yes – 40%
	No – 60%
Baseline ISI Score^1^	18 (16–20)

1 Displayed as mean (range).

2 Primary was not determined at time of diagnosis.

The CBT-I treatment phase occurred from March 25, 2024, to April 29, 2024. One participant withdrew from the study after the second CBT-I session and was included in the feasibility analysis but excluded from all other post-intervention analyses.

### 4.2 Primary endpoints – feasibility and acceptability

Feasibility metrics for attendance and participation were met. On an individual participant level, there was an average of 63% attendance (*N* = 5, range 40–80%). For the four participants who completed the intervention, attendance was 71% (*n* = 4, range 50–100%). On a group level, three of the initially enrolled participants attended four or more sessions (*N* = 5, 60%). Of the four participants who completed the intervention in its entirety, 75% attended four or more sessions. In the initially enrolled group, the median number of sessions attended was four, with participants attending 67% of all possible sessions, thereby meeting the feasibility benchmark. For those who completed the intervention, the median number of sessions attended was 4.5 (n = 4, range: 3–6) (Table 2).

**Table 2:** Acceptability of Virtual, Small-Group Format for CBT-I (N = 5).

Session Number	Number of Participants Attended	Number Responded Acceptable	Number Responded Unacceptable	Number Attended but Did Not Respond	Acceptability Rate of All Attendees (Number Acceptable / Number Attended)	Acceptability Rate of All Responders (Number Acceptable / Number Responded)
1	4	4	0	0	100.0%	100.0%
2	4	2	0	2	50.0%	100.0%
3	4	2	1	0	50.0%	66.7%
4	3	3	0	0	100.0%	100.0%
5	2	2	0	0	100.0%	100.0%
6	3	2	1	0	66.7%	66.7%
**Average Acceptability for All Sessions**					**77.8%**	**88.9%**

All subjects participated at least once during every session they attended, resulting in 100% participation. Internet connectivity issues only occurred during the second session. During that session, three participants had issues with logging into the virtual meeting (joining 10–18 min late). Furthermore, one participant left the meeting early. Despite these connectivity issues, all participants attended the session for >50% of the time.

Acceptability was also achieved for this trial. Every session had at least 50% acceptability of a virtual, small-group format in lieu of private, one-on-one or an in-person setting. Average acceptability for all sessions was 77.8% (N = 5, range 50%–100%). Of those who attended and responded to the acceptability questionnaire, average acceptability for all sessions was 88.9% (N = 5, range 66.7%–100%) (Table 2).

In the open-ended responses of the acceptability survey, participants were queried about what they liked and what could have been improved. Participants appreciated the sense of community that arose from the group and enjoyed being able to share their experiences and hear others' perspectives. Many found it more encouraging to tackle challenging sleep modifications alongside others. They also found the program and facilitator compassionate and informative. Examples of participant feedback included:

• “Feeling that I was not alone, that others trying to get help for same issues as mine. Good to hear voices other than my own to gain perspective on how others are managing sleep. Feel supported.”
• “Sharing our struggles/triumphs during the week with new sleep schedule. Sharing relaxation breathing technique together. Listening to each other's experiences helps.”
• “The very personal experience within a group. A compassionate facilitator who gave each of us plenty of attention and problem solving which added an additional level of perspectives. Hearing other experiences a great teaching experience.”
• “[The facilitator] is great! Very informative.”
• “All the sessions are so beneficial.”
• “Encouragement and help with anxiety that prevents me from sleeping. Schedule worry time.”
• “No judgement- others understand”

Regarding areas of improvement, some common themes included technical issues during the session or with the Insomnia Coach Explorer App, length of session being too long, and desire for more participants. The most common feedback regarding improvement was “nothing” to improve. Below are some descriptive examples of what participants thought needed to be improved:

• “My iPad did not allow me to see other participants but just got that resolved so looking forward to an even better experience.”
• “Had a tiny bit of trouble with the app. Did not dampen enthusiasm and involvement in this study. Was a privilege and have so much gratitude and appreciation for the people involved in this study.”
• “Would be nice if more people were participating in study.”
• “Sessions are too long. I would prefer a short session with a plan and not so much talking.”

### 4.3 Secondary endpoints – insomnia symptoms, quality of life, and sleep characteristics

**Fig. 2:**
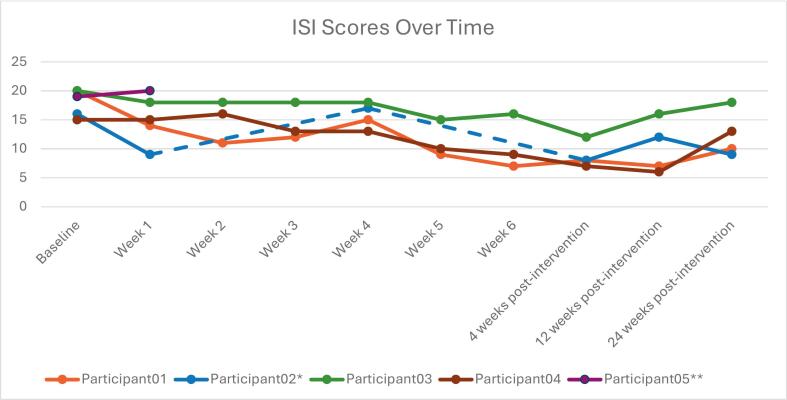

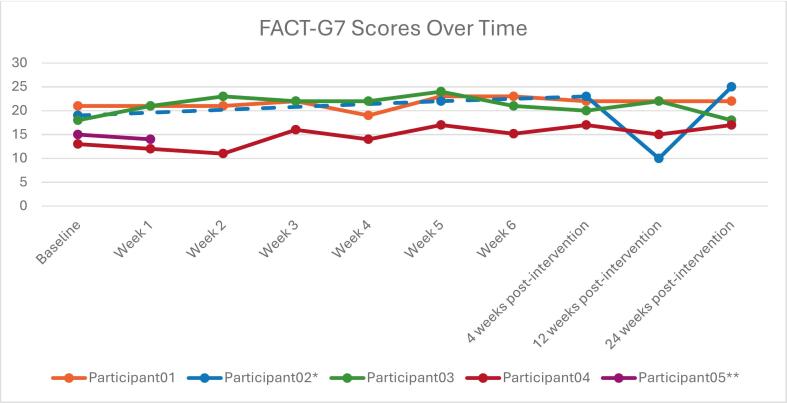

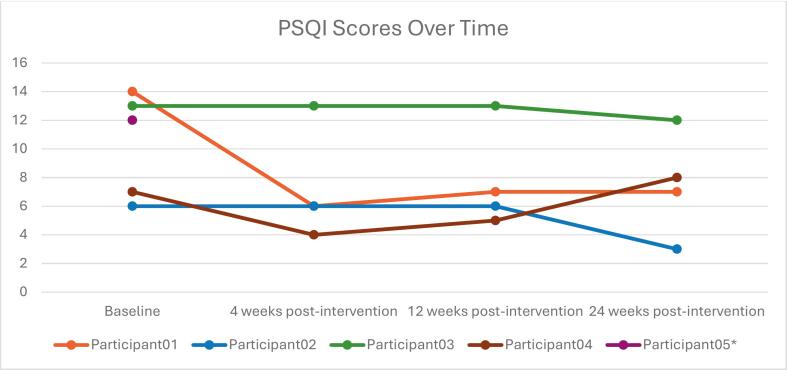
ISI, PSQI, and FACT-G7 Scores. a. ISI scores during the intervention and follow up *Participant02 did not complete assessment for week 2, 3, and 5. The dashed line connects the scores for the completed assessments. **Participant05 withdrew from study after week 1. b. FACTG7 scores during the intervention and follow up *Participant02 did not complete assessment for week 2, 3, and 5. The dashed line connects the scores for the completed assessments. **Participant05 withdrew from study after week 1. c. PSQI scores at baseline and during follow up *Participant05 withdrew from study after week 1.Caption: ISI, PSQI and FACT-G7 scores were trended over time. 2a. By week 5, ISI scores had significantly improved compared to baseline. 2b. FACT-G7 scores did not significantly change during the intervention or the follow up period. 2c. PSQI did not significantly change during the follow up period.

The results of ISI, PSQI, and FACT-G7 at baseline, throughout the intervention, and during the follow-up period are displayed in Fig. 2. By the fifth week of the intervention, mean ISI scores decreased by more than six points from baseline (11.47, 95% CI: 7.78–15.16). By the end of the intervention, the mean ISI scores were below the threshold for moderate clinical insomnia (ISI ≥ 15), and this improvement in ISI scores lasted throughout the follow-up period (Table 3).

**Table 3:** ISI and FACT-G7 scores during 6-week CBT-I program and post-intervention follow-up.

Assessment Scores	Baseline Mean (95% CI)	Week 1 Mean (95% CI)	Week 2 Mean (95% CI)	Week 3 Mean (95% CI)	Week 4 Mean (95% CI)	Week 5 Mean (95% CI)	Week 6 Mean (95% CI)	4 weeks post-intervention Mean (95% CI)	12 weeks post-intervention Mean (95% CI)	24 weeks post-intervention Mean (95% CI)
ISI	18.00 (15.09–20.91)	15.20 (12.01–18.39)	15.14 (11.45–18.83)	14.47^2^ (10.78-18.16)	16.29 (12.88–19.70)	11.47^2^ (7.78-15.16)	10.81^2^ (7.12-14.5)	9.29^2^ (5.88-12.7)	10.79^2^ (7.38-14.2)	13.04^2^ (9.63–16.45)
FACT-G7	17.20 (13.23–21.17)	17.17 (13.67–21.07)	17.92 (13.64–22.2)	19.59 (15.31–23.87)	17.92 (13.64–22.2)	21.02^2^ (17.09-24.95)	19.31 (15.03–23.59)	20.02 (16.09–23.95)	16.77 (12.84–20.7)	20.02 (16.09–23.95)
PSQ^1^I^1^	10.40 (8.87-14.93)							7.52 (3.65–11.38)	8.02 (4.15–11.88)	7.77 (3.90–11.63)

1 PSQI only collected at baseline and during follow-up.

2 p < 0.05 when compared to baseline, *p*-values not adjusted for multiplicity.

The mean PSQI scores did not change significantly during the study period (Table 3). However, Participant01 did have a clinically meaningful decrease in her score from 14 to 6, which persisted during the follow up period (Fig. 2c). There were no significant changes in FACT-G7 scores either during the 6-week program or the follow-up period (Table 3).

During the CBT-I intervention, participants completed their sleep diary via the Insomnia Coach Explorer App. In reviewing the trends of sleep outcomes, there was a significant decrease in the number of times awoken during the night from a baseline of 2.10, resulting in a decrease of 0.21 per week during the 6-week intervention (*p* = 0.021). There were also non-significant decreases in WASO and sleep latency. There was a non-significant increase in sleep efficiency. Finally, there was a non-significant decrease in total sleep time, which may be due to the goals of sleep restriction as part of the CBT-I program (Table 4).

**Table 4:** Sleep Outcomes Based on Sleep Diaries During the Intervention.

Sleep Outcomes	Baseline	Change per week during CBT-I intervention
Total sleep time (minutes)	361.08	−10.5
Wake after sleep onset (WASO) (minutes)	20.22	−2.24
Number of Times Awoken	2.10	-0.21^1^
Sleep Latency (minutes)	49.40	−3.99
Sleep Efficiency (%)	72.31	+1.82^1^

1 p < 0.05, p-values not adjusted for multiplicity.

## 5 Discussion

This feasibility study demonstrated that CBT-I delivered in a live, virtual, small-group format is feasible and acceptable for patients with gynecologic malignancies and is likely associated with improvements in insomnia symptoms. These findings align with prior studies that have demonstrated CBT-I to be an effective treatment for insomnia in oncology patients. This study extends emerging digital delivery models to gynecologic oncology patients, a population that has been largely underrepresented in behavioral sleep intervention research in spite of a high insomnia burden (Epstein and Dirksen, 2007; Espie et al., 2008; Fiorentino et al., 2010; Padron et al., 2022).

Despite scheduling challenges, four of the five participants enrolled in our study completed the intervention, yielding an 80% retention rate. Participants also demonstrated consistent in-session engagement. Some technical issues with connectivity and the sleep diary app did emerge but were quickly resolved by the research team. Therefore, technical issues were minimal and did not pose meaningful barriers to attendance or participation. These results met the predefined feasibility criteria and are comparable to the retention, attendance, and engagement rates reported in prior CBT-I trials in oncology populations (Savard et al., 2005; Espie et al., 2008; Padron et al., 2022). Furthermore, most participants perceived virtual, small-group CBT-I as acceptable. Their qualitative feedback was largely positive, consistent with earlier studies on digitally delivered CBT-I interventions (Ritterband et al., 2012; Hall et al., 2022; Loughan et al., 2023; Starling et al., 2024). Collectively, these findings suggest that remote, group-based CBT-I is a feasible and acceptable intervention for gynecologic oncology patients.

Access to CBT-I remains limited, but more recently, internet-based interventions are emerging as alternative delivery formats that may improve access to care (Bastien et al., 2004). Internet-delivered CBT-I was found to be non-inferior compared to in-person, group CBT-I for patients in the general population. Both methods yielded sustained improvements in insomnia severity, sleep efficiency, sleep latency, and sleep quality in a general population (Blom et al., 2015). Additionally, studies of internet-based CBT-I in oncology patients have reported similar improvements in sleep quality (Ritterband et al., 2012; Starling et al., 2024; Clara et al., 2025). However, limited research has been conducted to directly compare in-person, individualized CBT-I with a live, online, group format. In one randomized controlled trial of breast cancer patients, therapist-led CBT-I produced greater improvements in sleep compared to pre-recorded, self-administered CBT-I and the no-treatment control (Savard et al., 2014). Furthermore, a cost-effectiveness model demonstrated that in person CBT-I was 5.5 times greater in cost compared to the self-administered format (Savard et al., 2021). While this study's design does necessitate the cost of a CBT-I therapist, the cost savings for participants due to transportation and online material delivery are still likely meaningful.

Studies evaluating the efficacy of CBT-I delivered in a live, virtual group format for oncology patients are limited. Some prior trials have demonstrated improvements in ISI, a finding that was also observed in this study (Hall et al., 2022; Loughan et al., 2023). All of the participants in this study began with an ISI score of ≥15, which is the threshold for moderate to severe insomnia. By the end of this program, most participants' ISI scores had decreased to below 15, an effect that persisted throughout the follow-up period. The benefits of CBT-I appear to be durable, as multiple studies have shown continued improvement for up to 12 months after the intervention (Morin et al., 1999; Savard et al., 2005; Espie et al., 2008). Together, these results suggests that this delivery model may preserve the therapeutic benefits of therapist-led CBT-I while improving accessibility.

Improvements in insomnia severity were also reflected in several sleep characteristics observed during this intervention. Multiple studies of group CBT-I and internet-based interventions focusing on cancer patients demonstrated improvements in sleep variables such as sleep onset latency (SOL), sleep efficiency (SE), total wake time (TWT), and wake after sleep onset (WASO) compared to control (Savard et al., 2005; Epstein and Dirksen, 2007; Dirksen and Epstein, 2008; Espie et al., 2008; Ritterband et al., 2012; Clara et al., 2025). Consistent with previous studies, participants in this study experienced a significant reduction in the number of nocturnal awakenings during the intervention. While changes in WASO, SOL, and SE were not statistically significant, these measures exhibited a trend toward improvement indicating improved sleep continuity. A non-significant decrease was also observed in total sleep time, which may be attributed to the sleep restriction component of CBT—I. Improvements in these sleep characteristics seen in prior studies, as well as this one, suggest that group CBT-I can favorably influence multiple aspects of sleep.

Although improvements in insomnia severity were seen, this study did not find any significant changes in quality-of-life outcomes, as measured by the PSQI and FACT-G7. Prior studies evaluating CBT-I in oncology populations have reported mixed findings with respect to quality-of-life outcomes, including fatigue, depression, and anxiety. While some studies observed significant improvements of quality of life, others reported more modest or non-significant changes (Dirksen and Epstein, 2008; Ritterband et al., 2012; Starling et al., 2024; Clara et al., 2025). The varying effects of CBT-I on quality of life are likely due in part to the multifactorial reasons for insomnia. The non-significant changes in quality of life seen in this study may reflect the varied drivers of quality of life among gynecologic oncology patients, including ongoing treatment effects, pain, fatigue, psychological distress, and access to support.

While insomnia affects 29% of patients with gynecologic cancers, it can be as high as 60% for patients with ovarian cancer (Davidson et al., 2002; Price et al., 2009). In this study, three out of five participants had ovarian, fallopian tube, or peritoneal carcinomas. It is not surprising that the rates of insomnia are so high in this population. Ovarian cancer is often diagnosed at late stages, and patients require cancer-directed therapies for prolonged durations. Insomnia can stem from not only the anxiety related to receiving this diagnosis, but also chemotherapy itself, which leads to more daytime sleepiness and worse sleep quality, often resulting in a poorer quality of life (Clevenger et al., 2013). (Savard et al., 2005, Savard et al., 2014, Savard et al., 2021; Epstein and Dirksen, 2007; Espie et al., 2008; Fiorentino et al., 2010; Matthews et al., 2014) Despite the high insomnia burden in patients with gynecologic cancer, there are no insomnia screening guidelines specific to this patient population. The NCCN recommends screening for sleep disorders for all cancer patients at regular interval and at least annually, and particularly when there is a change in clinical status or treatment (NCCN Surviv. Guidel.). Also, few studies have evaluated effective treatments for insomnia in gynecologic oncology patients. A randomized controlled trial of in-person individualized CBT-I vs psychoeducation control for patients with gynecologic cancer found that CBT-I improved sleep efficiency, sleep latency, total wake time, and wake after sleep onset (Padron et al., 2022). However, there are no published treatment trials on the efficacy of virtual, small-group CBT-I in gynecology cancer patients at the time of this publication, so this study contributes to the data.

In addition to addressing a patient population that has been largely underrepresented in CBT-I research, this study showed feasibility and acceptability of implementing a live, virtual, small-group CBT-I intervention, the gold-standard treatment for insomnia. The retention and participant engagement throughout this study support the practicality of this approach. The signals of improvements in ISI scores and sleep outcomes, and the participants' positive feedback, further demonstrate that this model can provide the benefits of both therapist-led interventions and group therapy. Moreover, this intervention removes the cost of transportation and distributes the cost of a CBT-I session across multiple patients, making it a more cost-effective option in comparison to traditional CBT—I. Finally, the inclusion of a longitudinal follow-up period in this study allowed for assessment of the durability of insomnia-related outcomes.

Although this study has several strengths, the results should be interpreted in the context of several limitations. First, the small sample size substantially limits the robustness of the findings as it reduces statistical power, limits the precision of effect estimates, and increases the probability that observed outcomes may not be representative of the broader gynecologic oncology population. Second, the absence of a control group precludes evaluation of comparative effectiveness. Further research is needed to determine the effects of this model compared with traditional CBT-I in patients with gynecologic cancer. Third, sleep outcomes were assessed using self-reported metrics, which may be subject to reporting bias and measurement errors. Finally, the inclusion of only English-speaking participants and the racial homogeneity of the study population further limit the generalizability of these findings. Despite these limitations, this pilot study offers valuable insights that will guide future research. Future studies should expand recruitment to include more diverse patient populations and evaluate the feasibility, acceptability, and effectiveness of implementing culturally and linguistically adapted CBT-I in this digital format. These efforts will be critical for improving generalizability and addressing disparities in CBT-I access. Further work is also needed to optimize the virtual, group format and to compare its effectiveness with other delivery models, including traditional CBT-I and in-person group CBT—I. Finally, assessing the cost-effectiveness, optimal frequency and duration of CBT-I sessions, and mode of delivery of this intervention will be important for supporting its implementation.

## CRediT authorship contribution statement

**Abigail Low:** Writing – original draft, Project administration, Investigation, Formal analysis, Data curation. **Jena Cooreman:** Writing – original draft, Supervision, Resources, Project administration, Methodology, Investigation, Data curation, Conceptualization. **Michelle L. Dossett:** Writing – review & editing, Writing – original draft, Validation, Supervision. **Katherine Van Deventer:** Writing – review & editing, Project administration, Methodology, Investigation, Data curation, Conceptualization. **Matthew Ponzini:** Writing – original draft, Methodology, Investigation, Formal analysis. **Machelle Wilson:** Writing – original draft, Methodology, Formal analysis, Data curation, Conceptualization. **Codi Cole:** Writing – review & editing, Methodology, Data curation, Conceptualization. **Rachel Ruskin:** Writing – review & editing, Resources, Project administration, Methodology, Investigation. **Gary Leiserowitz:** Writing – review & editing, Project administration, Methodology, Investigation. **Rebecca Brooks:** Writing – review & editing, Resources, Project administration, Investigation. **Nancy Nguyen:** Writing – review & editing, Resources, Project administration, Investigation. **Melissa Pham:** Writing – review & editing, Resources, Project administration, Investigation. **Hui Chen:** Writing – review & editing, Writing – original draft, Supervision, Project administration, Methodology, Investigation, Funding acquisition, Formal analysis, Data curation, Conceptualization.

## Funding

This work was supported by the National Institute of Health, Building Interdisciplinary Research Careers in Women's Health at 10.13039/100007707University of California Davis through Grant Number: 5K12HD051958.

## Declaration of competing interest

The authors declare the following financial interests/personal relationships which may be considered as potential competing interests: Hui Chen reports financial support was provided by National Institutes of Health Office of Research on Women’s Health. Co-author receives royalties from UpToDate for a topic unrelated to this work - MLD If there are other authors, they declare that they have no known competing financial interests or personal relationships that could have appeared to influence the work reported in this paper.
